# Row selection in remote sensing from four-row plots of maize and sorghum based on repeatability and predictive modeling

**DOI:** 10.3389/fpls.2023.1202536

**Published:** 2023-06-20

**Authors:** Seth A. Tolley, Neal Carpenter, Melba M. Crawford, Edward J. Delp, Ayman Habib, Mitchell R. Tuinstra

**Affiliations:** ^1^ Department of Agronomy, Purdue University, West Lafayette, IN, United States; ^2^ Analytics and Pipeline Design, Bayer Crop Science, Chesterfield, MO, United States; ^3^ Lyles School of Civil Engineering, Purdue University, West Lafayette, IN, United States; ^4^ School of Electrical and Computer Engineering, Purdue University, West Lafayette, IN, United States

**Keywords:** remote sensing, high-throughput phenotyping, RGB, lidar, hyperspectral, UAV, plot trimming, border effect

## Abstract

Remote sensing enables the rapid assessment of many traits that provide valuable information to plant breeders throughout the growing season to improve genetic gain. These traits are often extracted from remote sensing data on a row segment (rows within a plot) basis enabling the quantitative assessment of any row-wise subset of plants in a plot, rather than a few individual representative plants, as is commonly done in field-based phenotyping. Nevertheless, which rows to include in analysis is still a matter of debate. The objective of this experiment was to evaluate row selection and plot trimming in field trials conducted using four-row plots with remote sensing traits extracted from RGB (red-green-blue), LiDAR (light detection and ranging), and VNIR (visible near infrared) hyperspectral data. Uncrewed aerial vehicle flights were conducted throughout the growing seasons of 2018 to 2021 with data collected on three years of a sorghum experiment and two years of a maize experiment. Traits were extracted from each plot based on all four row segments (RS) (RS1234), inner rows (RS23), outer rows (RS14), and individual rows (RS1, RS2, RS3, and RS4). Plot end trimming of 40 cm was an additional factor tested. Repeatability and predictive modeling of end-season yield were used to evaluate performance of these methodologies. Plot trimming was never shown to result in significantly different outcomes from non-trimmed plots. Significant differences were often observed based on differences in row selection. Plots with more row segments were often favorable for increasing repeatability, and excluding outer rows improved predictive modeling. These results support long-standing principles of experimental design in agronomy and should be considered in breeding programs that incorporate remote sensing.

## 1 Introduction

Maize (*Zea mays* L.) and sorghum (*Sorghum bicolor* (L.) Moench) are among the most important crops in the world that are utilized for food, animal feed, biofuels, and other applications. These crops are desirable because of their C4 photosynthetic pathways that enable greater photosynthetic potential in hot, dry environments. These crops are likely to be of increasing global importance in the coming years and continued crop improvement are needed to improve food security (Ray et al., 2013).

Remote sensing using sensors attached to uncrewed aerial vehicles (UAV) enables in-season field measurements with high spatial and temporal resolutions necessary for small-plot research (Maes and Steppe, 2019; Ravi et al., 2019; Masjedi et al., 2020). Remote sensing has been used for plant phenotyping for a number of crops including maize (Zaman-Allah et al., 2015; Pugh et al., 2018; Anderson et al., 2019; Anche et al., 2020), sorghum (Watanabe et al., 2017; Pugh et al., 2018; Masjedi et al., 2020; Yang K. et al., 2021), soybean (Moreira, 2020), and wheat (Tattaris et al., 2016; Crain et al., 2018; Hassani et al., 2023). Various sensors are now widely flown on a UAV including RGB (red-green-blue) cameras, multispectral/hyperspectral sensors, and LiDAR (light detection and ranging) units. With an onboard Global Navigation Satellite System/Inertial Navigation System (GNSS/INS) and system calibration, data from these sensors can be georeferenced directly with high spatial accuracy (Ravi et al., 2019). These sensors collect large amounts of data that provide opportunities for the measurement, estimation, or prediction of a wide range of attributes such as grain yield, biomass productivity, leaf area index, canopy cover, plant counts, nitrogen content, and disease detection (Yang et al., 2007; Zarco-Tejada et al., 2012; Mathews and Jensen, 2013; Ribera et al., 2017; Li et al., 2018; Toledo et al., 2022; Hassani et al., 2023).

In maize and sorghum breeding programs, experimental hybrids are evaluated in field trials, and the genotypes with the most desirable combinations of traits are selected and advanced in the respective breeding programs. Nevertheless, traditional hybrid testing programs are time consuming and expensive; creating a phenotyping bottleneck for progress in crop improvement (Cobb et al., 2013; Yang et al., 2017). Phenotyping by remote sensing has the potential to relieve the phenotyping strain on breeding programs by reducing the time-consuming and laborious nature of low-throughput phenotyping while simultaneously increasing the throughput on the number of plots that can be evaluated (Furbank and Tester, 2011; Araus and Cairns, 2014; Araus et al., 2018). However, given the observation geometry and sensor technologies, certain traits cannot be computed directly from remotely sensed data, as with traditional phenotyping methods.

High-throughput phenotyping by remote sensing enables the evaluation of more plots and can improve genetic gain in plant breeding. More genotypes can be evaluated in the same land area by either decreasing the plot size or by maintaining plot size and increasing land area and consequently cost. Often, this tradeoff is balanced in a breeding program by using small plots in preliminary trials and larger plots in advanced breeding trials where yield is a primary trait of interest (Acquaah, 2012). A common agronomic practice is to make phenotyping measurements on the inner rows of a multi-row plot to minimize border effects between competing genotypes. The border row effects have been well defined and as a result these outer plot rows are harvested separately or ignored in yield trials (Bird, 1929; Genter, 1958; Gomez, 1972, Kramer et al., 1982; Bowman, 1989; Petersen, 1994; Ceccarelli and Grando, 1996; Reynolds & Braun, 2022) (**Supplemental Figure 1**). Zhang et al. (2019) found that soybean yield was best predicted where hyperspectral data was analyzed for a subset of 20 to 80% of the plot area. More work is needed to understand the border effect on remote sensing traits and their use in breeding programs.

Trait repeatability is an important factor in plant breeding programs (Bernardo, 2014). Repeatability is defined as the signal to noise ratio for phenotypic measurements, indicating the relative importance of genetic effects in comparison to environmental effects in phenotypic observations. Proper experimental design including replication, blocking, randomization, and plot size all influence the trait repeatability. Barmeier and Schmidhalter (2016) demonstrated that high-throughput spectral phenotyping can be used in small-plot research comprising single or multiple rows. However, no study has evaluated the impact of row selection or plot trimming on the repeatability of remote sensing traits. Additionally, a primary goal of in-season remote sensing is to predict end-season yield (biomass or grain). Selection of rows from remote sensing data products could be an important consideration impacting the results of predictive modeling.

In this study, experimental hybrids of maize and sorghum were evaluated in four-row plots in field trials from 2018 to 2021. Remote sensing flights were conducted with RGB, LiDAR, and VNIR (visible near infrared) hyperspectral sensors throughout the growing season. Biomass and grain yield were evaluated in sorghum and maize, respectively, at the end of the growing seasons. The objectives of this experiment were to (1) determine the impact of row selection on repeatability based on remote sensing traits, (2) compare repeatability of remote sensing-based traits with end-trimming and no trimming, and (3) demonstrate the impact of row selection on end-season biomass and grain yield prediction.

## 2 Materials and methods

### 2.1 Field experiment and germplasm

**Figure 1:**
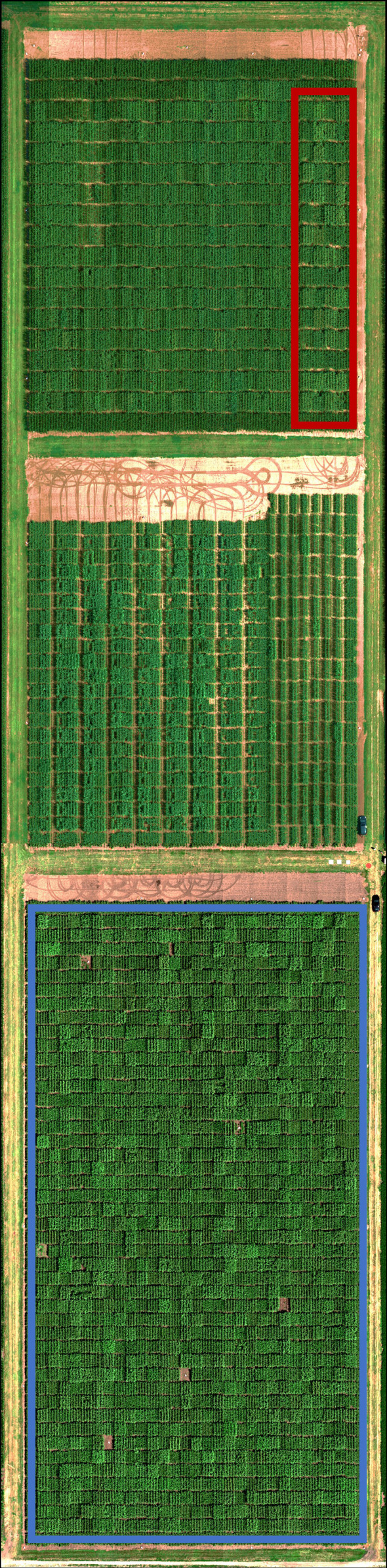
Hyperspectral orthophoto colored as red-green-blue showcasing the 2020 sorghum (large, blue box) and maize (small, red box) experiments used in this study.

Field trials were conducted from 2018 to 2021 in West Lafayette, Indiana, US at the Agronomy Center for Research and Education at Purdue University (40°28’37.18”N, 86°59’22.67”W). The experiments were planted as four-row plots with a length of 3.05 m by 3 m with 76 cm row spacing (**Figure 1**). Alleys were 76 cm in length between ranges. Nutrients, herbicides, and insecticides were applied according to best agronomic practices to not limit plant growth and development in these experiments. In each study, the experiments were planted in a crop rotation with soybean.

In 2018, 2019, and 2020, 619 inbred lines from the sorghum diversity panel were assessed for their testcross hybrid performance with ATx623 (sorghum reference genome). This experiment was planted on May 8, 2018; June 4, 2019; and May 12, 2020 with a consistent seeding rate of 220,000 seeds ha^-1^. On June 7, 2018, 28% liquid UAN was applied at 16 g N m^-2^. In October 2018, 34 g m^-2^ of potash (0-0-60) and 1,121 g m^-2^ of lime for the 2019 growing season. Additionally, 18 g N m^-2^ of anhydrous ammonia was applied on May 9, 2019. On April 6, 2020, 18 g N m^-2^ of anhydrous ammonia was applied. The sorghum experiments followed a randomized complete block design with 2 replications. End-season biomass was harvested from rows two and three of each plot on August 14, 2018; September 12, 2019; and August 19, 2020 using a Wintersteiger Cibus Biomass Harvester (Wintersteiger Inc., Salt Lake City, UT, USA). Moisture content was determined by sampling ~500 g of chopped biomass from each plot from which fresh weight and dry weight (after samples were dried for ten days at 72°C) were recorded. Moisture content was used to adjust biomass from fresh weight to dry weight.

In 2020 and 2021, a maize field experiment was grown comprised of ten temperate and ten tropical inbred lines evaluated for their testcross hybrid performance with PHP02. This experiment was planted on May 12, 2020 and May 23, 2021 at a population of 74,000 seeds ha^-1^ using a randomized complete block design with 3 replications. On April 6, 2020, 18 g N m^-2^ of anhydrous ammonia was applied. On April 14, 2021, 28% liquid UAN was applied at 16.25 g N m^-2^. Grain yield was harvested from rows two and three of each plot on October 14, 2020 and October 21, 2021 using a Kincaid plot combine (Kincaid 8-XP, Haven KS, USA) with grain yields adjusted to 15% moisture.

### 2.2 Sensors

A DJI M600 pro equipped with an onboard Applanix APX-15 GNSS/INS unit, which allowed for direct-georeferencing, and integrated RGB, LiDAR, and VNIR sensors, was used in each of the studies. Boresight calibrations were performed for each sensor for co-alignment of the sensors and flight dates as described by Habib et al. (2018); Ravi et al. (2018), and LaForest et al. (2019). RGB data were collected using a Sony Alpha 7R digital mirrorless camera with a Sony Sonnar T* FE 35 mm lens. The Sony Alpha 7R camera features a full-frame 36.4 MP sensor delivering high-resolution images. Georeferenced orthophotos were generated using a structure-from-motion strategy introduced by He et al. (2018) and Hasheminasab et al. (2020). LiDAR data were collected with a Velodyne VLP-16 instrument, which has 16 beams and a 360-degree horizontal field of view with a maximum range of 100 m. It can scan up to 300,000 points second^-1^ with a typical accuracy of ±3cm. VNIR data were collected with a Headwall Photonics Nano-Hyperspec, a hyperspectral push-broom scanner collecting data in 272 spectral bands at 2.2 μm band^-1^ from 400 nm to 1000 nm. It has 640 spatial channels with a7.4μm pixel pitch. Spectral targets calibrated using re Spectra Vista SVC 1024i were used in post-processing to convert from radiance to reflectance via the Empirical Line Method. VNIR orthophotos were obtained using the digital surface model from the georeferenced LiDAR point cloud through the approach developed by Lin and Habib (2021).

### 2.3 Flight information

Flight information including date, growing degree days (GDD), and sensor data collection is given in **Table 1** and **Supplemental Tables 1, 2**. Flights were conducted at an altitude of ~40 m with a speed of ~4 m s^-1^ for a ground sampling distance of ~1 cm in RGB orthophotos and ~4cm in VNIR orthophotos. Generally, flights were conducted around solar noon on clear days with little wind. GDD for each day was calculated using the formula, GDD = [(T_max_+T_min_)/2]-10. When the maximum and minimum temperatures were greater than 30°C or less than 10°C, then T_max_ and T_min_ were set to 30°C and 10°C, respectively (Gilmore and Rogers, 1958). Flights were organized across years based on GDD into vegetative (0-650 GDD), flowering (650-900 GDD), and grain filling (900+GDD) growth stages. These GDD windows were based on the average beginning and end of flowering across the years of the maize and sorghum experiments, although there were a few late maturing sorghum genotypes that did not begin flowering until later in the growing season each year.

**Table 1:** Number of flights with RGB (red-green-blue), LiDAR (light detection and ranging), and VNIR (visible near infrared hyperspectral) at each growth stage (vegetative, 0 – 650 GDD; flowering, 650 – 900 GDD; grain filling (900+ GDD) in maize and sorghum experiments from 2018 – 2021.

Crop	Growth Stage	Sensor Flights			
		RGB	LiDAR	VNIR	
**Maize**	**Vegetative**	8	6	6
	**Flowering**	6	4	3
	**Grain Filling**	8	6	6
**Sorghum**	**Vegetative**	9	7	5
	**Flowering**	7	6	4
	**Grain Filling**	6	5	6

### 2.4 Remote sensing traits

Remote sensing traits were collected from RGB, LiDAR, and VNIR sensors and were extracted for each row segment (RS) of a plot 2D canopy cover (CC) was estimated from RGB imagery as the ratio of the number of pixels attributed to vegetative material, based on thresholds in the HSV (Hue, Saturation Value) color space, to the total number of pixels in a RS (Chen, 2019) and 3D canopy cover by LiDAR as the number of points above the 10^th^ (CC10) percentile divided by the total number of points in each RS (Masjedi et al., 2020). LiDAR was also used to quantify variation in height and plot volume. Height was determined at the 95^th^ (Height 95%) percentile of the nonground points. Plot volume was estimated by assigning cell sizes 8 cm x 8 cm to each plot. Within each grid, height was calculated as the average of the 95^th^ percentile height and the minimum height of nonground points. Average height was multiplied by the cell size to estimate the volume of vegetation in a plot. Vegetation indices were calculated from the plant material in hyperspectral orthophotos where the ground was masked using thresholds of vegetation indices (**Table 2**).

**Table 2:** Summary of vegetation indices obtained from the VNIR-hyperspectral sensor used in predictive modeling.

Abbreviation	Index Name	Formula	Reference
Carte1	Carter Index 1	R_695_/R_420_	Carter (1994)
GNDVI	Green NDVI	(R_750_ - R_550_)/(R_750_ + R_550_)	Gitelson et al., 1996
mRENDVI	Modified Red Edge Simple NDVI	(R_750_ - R_705_)/(R_750_ + R_705_ - 2R_445_)	Sims and Gamon (2002)
NDVI	Normalized Difference Vegetation Index	(R_750_ - R_705_)/(R_750_ + R_705_)	Gitelson and Merzlyak (1994)
OSAVI	Optimized Soil Adjusted Vegetative Index	(1 + 0.16)(R_800_ - R_670_)/(R_800_ + R_670 + _0.16)	Rondeaux et al. (1996)
PRI	Photochemical Reflectance Index	(R_531_ - R_570_)/(R_531_ + R_570_)	Gamon et al. (1992)
PSRI	Plant Senescence Reflectance Index	(R_680_ - R_500_)/R_750_	Merzlyak et al. (1999)
SR800680	Simple Ratio Index	R_800_/R_680_	Sims and Gamon (2002)
SR700670	Simple Ratio Index	R_700_/R_670_	McMurtrey et al. (1994)
VOG1	Vogelmann Red Edge Index	R_740_/R_720_	Vogelmann et al. (1993)

### 2.5 Row selection and plot trimming

**Figure 2:**
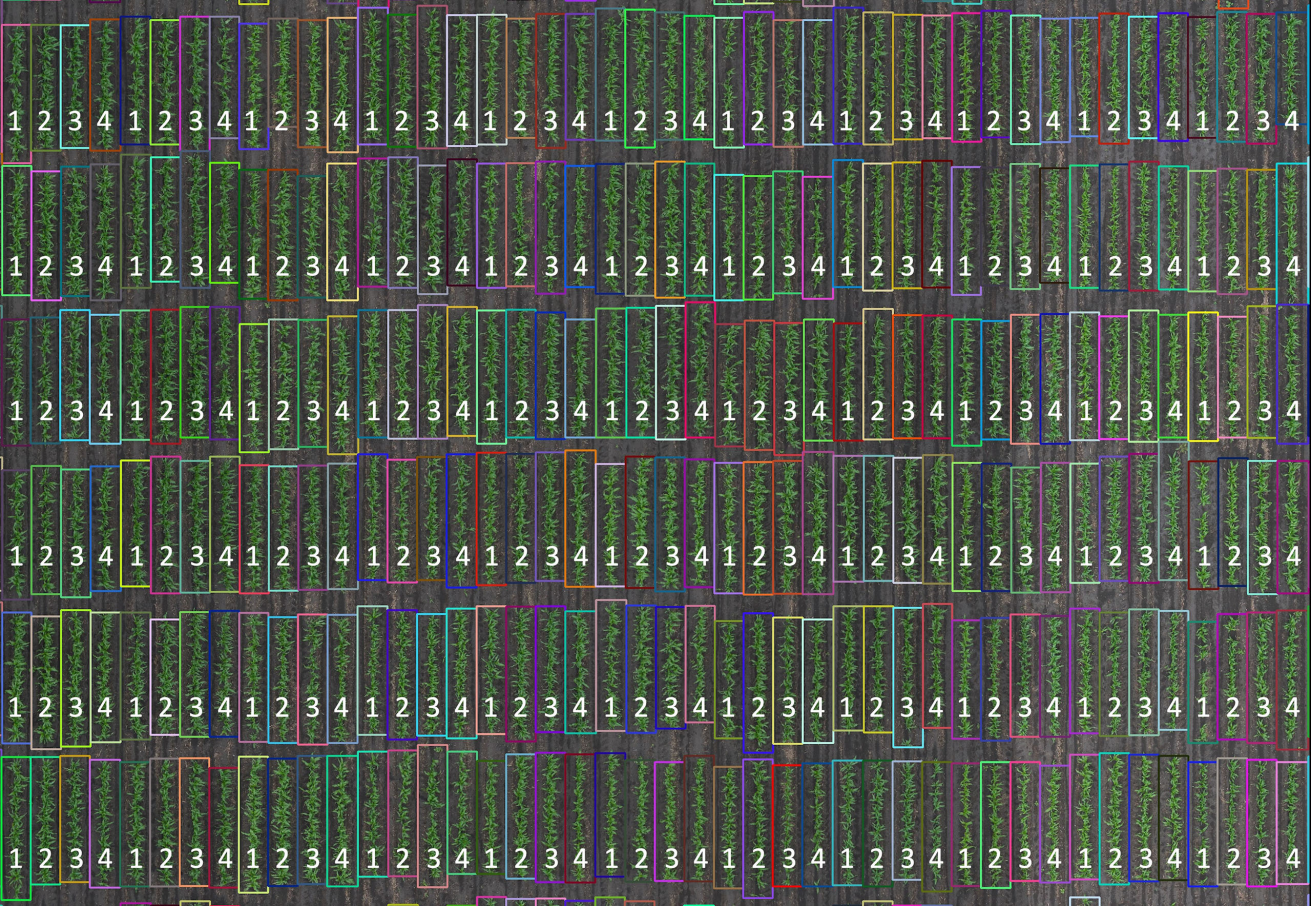
Row segmentation from a section of the sorghum experiment in 2018. Plots were planted as four-row plots. Row segments (1, 2, 3, and 4) within each plot were individually segmented. Row segment length was automatically determined based on the proximal and terminal ends of the plants in the individual row segments. Plot trimming was performed by removing 40 cm from the top and bottom of each row segment.

Row segmentation was performed as described by Yang et al. (2021a) to enclose the vegetation in rectangles. Briefly, the intersections of vertical and horizontal lines result in a grid of N x M coordinates where N is the number of ranges in the field and M is the number of rows. These grids result in bounding boxes around each individual RS of each plot in an early season RGB orthophoto (**Figure 2**). Bounding boxes do not all have exactly the same size because of variations in the location of emerging plants at the ends of the row segment. The plot means of a given remote sensing trait can be computed from all four RS (RS1234), the inner RS (RS23), the outer RS (RS14), and individual RS (RS1, RS2, RS3, RS4). For this study, plots were trimmed by removing 40 cm from the two ends of the RS boundary (**Supplemental Figure 2**).

### 2.6 Repeatability

Broad-sense heritability was calculated on an entry-mean basis (*H^2^*) to estimate repeatability of representative geometric and spectral remote sensing traits including: CC, CC10, plant height 95^th^ percentile (Height 95^th^), plot volume, and the normalized difference vegetation index (NDVI) (Nyquist and Baker, 1991; Piepho and Möhring, 2007). Variance components were estimated through restricted maximum likelihood (REML) from Equation 1 using R package ‘lme4’ (Bates et al., 2015). As remote sensing traits were observed on different dates across years and experiments, variance components were predicted for each year individually using the following model:


(1) $$Y_{ij}=\;µ\;+\;H_{i}\;+\;R_{j}\;+\;\varepsilon_{ij}$$

where Y*_ij_* is the phenotypic measurement of the *i^th^* hybrid in the *j^th^* rep. Components of the model include *µ* that represents the grand mean, *H_i_* as the random effect of the *i^th^* hybrid, *R_j_* as the fixed effect of the *j^th^* replicate, and ϵ*_ij_* as the residual error for the *i^th^* hybrid in the *j^th^* rep. Variance components for the random effects, hybrid and error, were estimated from Equation 1 and used to evaluate repeatability using Equation 2.


(2) $$H^{2}=\;\frac{\sigma_{H}^{2}}{\sigma_{H}^{2}\;+\;\frac{\sigma_{\varepsilon }^{2}}{rep}}$$

where *H^2^* represents repeatability of a given trait. Hybrid and error variance components are denoted by $\sigma_{H}^{2}$ and $\sigma_{\varepsilon }^{2}$ , respectively. The number of replications (2, sorghum; 3, maize) were *rep* in Equation 2.

### 2.7 Predictive modeling

Classical machine learning models including Support Vector Regression (SVR) and Partial Least Squares Regression were evaluated, and SVR was selected for further analysis based on its predictive performance as previously shown in Masjedi et al. (2020). Nevertheless, the goal of this study was to understand the impact of row selection and plot trimming on repeatability and predictive models, not to develop an optimum predictive model. SVR is a non-parametric regression technique with no statistical assumptions. SVR transforms the original feature space to find a linear hyperplane in a higher dimension for predictive modeling (Cristianini and Shawe-Taylor, 2000). Optimal values of hyperparameters sigma and cost were determined through cross-validation in a grid search. Parameters evaluated included sigma (0.001, 0.001, 0.01, 0.1) and cost (10, 50, 100, 150, 200, n) where n is the number of features used in the model (Masjedi, 2020). The correlation (*r*) of predicted yield with known yield in the testing set was used to assess model performance. SVR models were developed in R package ‘caret’ (Kuhn, 2008) implementing the model from R package ‘kernlab’ (Karatzoglou et al., 2004).

Remote sensing traits throughout the growing season were standardized (centered and scaled) and used to predict end-season biomass in sorghum and end-season grain yield in maize. In sorghum, 10-fold cross-validation repeated 100 times was used to assess model accuracy. In maize, 3-fold cross-validation repeated 500 times was used to assess model accuracy. An equal number of temperate and tropical germplasms were selected in each fold of cross validation to ensure similar population structures of training and testing datasets in maize. Plots of the same genotype from different replicates were considered as unique entries, and this was not a factor controlled in cross validation. Maize was repeated more than sorghum because of computational efficiency of the smaller experiment and to improve the precision of the prediction accuracy estimates.

### 2.8 Statistical analysis

Statistical analyses were performed in R (R Core Team, 2022). For the repeatability of a remote sensing trait from different dates and years, data were grouped based on GDD for degrees of freedom in analysis of variance (ANOVA). Flights were grouped as from 0 to 650 GDD representing vegetative growth stage, 0 to 900 GDD for flowering growth stage, and greater than 900 GDD for grain filling growth stage. Multiple flights in each growth stage interval in each year provide a robust dataset to perform statistical analyses (**Table 1**). ANOVA was used to determine the presence of significant interaction effects between year, row selection, and plot trimming in maize and sorghum. Where there was a significant difference between year, row selection, plot trimming, or the interaction effects in ANOVA results, a Least Significant Difference Test in R package ‘agricolae’ (de Mendiburu, 2021) was used to determine which treatments were significantly different at *ρ*< 0.05. Remote sensing data, yield data, and R code used for this study are available at the Purdue University Research Repository (10.4231/PF9S-4G38).

## 3 Results

### 3.1 Impact of row selection and plot trimming on repeatability

The repeatability of remote sensing traits was evaluated in multi-year sorghum (**Table 3**) and maize (**Table 4**) experiments. The average repeatability of all remote sensing traits was 0.84 in maize and 0.67 in sorghum. The repeatability values of remote sensing traits increased at later stages of development. The average repeatability values in sorghum were 0.54, 0.71, and 0.76 during the vegetative, flowering, and grain filling growth stages respectively. The average repeatability values in maize similarly increased at later growth stages with values of 0.81, 0.84, and 0.87 during the vegetative, flowering, and grain filling growth stages, respectively. Plot trimming and interaction effects involving plot trimming never resulted in a significant change in repeatability in either maize or sorghum.

**Table 3:** Repeatability of remote sensing traits from RGB (red-green-blue), LiDAR (light detection and ranging), and VNIR (visible near infrared hyperspectral) sensors in sorghum experiments from 2018, 2019 and 2019.

	Vegetative (0 - 650 GDD)					Flowering (650 - 900 GDD)					Grain Filling (900+ GDD)				
	CC^R^	CC 10%^L^	Height 95%^L^	Plot Volume^L^	NDVI^V^	CC^R^	CC 10%^L^	Height 95%^L^	Plot Volume^L^	NDVI^V^	CC^R^	CC 10%^L^	Height 95%^L^	Plot Volume^L^	NDVI^V^
Mean	0.42	0.37	0.71	0.65	0.56	0.4	0.48	0.94	0.95	0.8	0.56	0.59	0.92	0.92	0.83
															
Sorghum 2018	0.3 b						0.47 b	0.93 b	0.94 b	0.72 c		–		–	0.8 b
Sorghum 2019	0.5 a						0.46 b	0.96 a	0.95 a	0.81 b		0.64 a		0.92 b	0.83 ab
Sorghum 2020	0.53 a						0.58 a	0.94 ab	0.94 b	0.85 a		0.54 b		0.93 a	0.84 a
															
RS1234	0.47 a					0.45 a	0.6 a	0.97 a	0.96 a	0.84 a		0.69 a	0.96 a	0.95 a	0.88 a
RS23	0.45 ab					0.45 a	0.56 ab	0.98 a	0.96 a	0.83 b		0.68 ab	0.97 a	0.95 a	0.87 ab
RS14	0.43 abc					0.38 b	0.49 cd	0.93 b	0.94 b	0.82 c		0.57 bc	0.91 b	0.92 b	0.84 b
RS1	0.36 d					0.3 c	0.38 e	0.87 c	0.93 bc	0.77 e		0.48 c	0.84 c	0.88 c	0.74 c
RS2	0.42 abc					0.43 ab	0.51 bc	0.97 a	0.96 a	0.8 d		0.6 ab	0.96 a	0.94 ab	0.83 b
RS3	0.41 bc					0.4 ab	0.44 de	0.97 a	0.96 a	0.8 d		0.63 ab	0.96 a	0.94 ab	0.85 ab
RS4	0.4 cd					0.39 ab	0.39 e	0.9 b	0.91 c	0.74 f		0.47 c	0.87 c	0.88 c	0.78 c
															
Trim ANOVA	0.722 NS^†^	0.87 NS	0.993 NS	0.936 NS	0.822 NS	0.294 NS	0.169 NS	0.906 NS	0.835 NS	0.09 NS	0.939 NS	0.575 NS	0.87 NS	0.727 NS	0.322 NS
Year ANOVA	<0.001 ***	0.403 NS	0.216 NS	0.244 NS	0.541 NS	0.12 NS	<0.001 ***	<0.001 ***	0.04 *	<0.001 ***	0.984 NS	0.002 **	0.518 NS	0.011 *	0.025 *
RS ANOVA	<0.001 ***	0.844 NS	0.901 NS	0.83 NS	0.885 NS	<0.001 ***	<0.001 ***	<0.001 ***	<0.001 ***	<0.001 ***	0.987 NS	0.002 **	<0.001 ***	<0.001 ***	<0.001 ***
RS x Y ANOVA	0.292 NS	1 NS	1 NS	1 NS	1 NS	0.355 NS	0.014 *	0.198 NS	0.492 NS	0.002 **	0.907 NS	0.999 NS	0.389 NS	1 NS	0.712 NS

Significant differences were declared using ANOVA between plot trimming (Trim) techniques, years (Y), row segments (RS), and interaction (RS x Y) effects. Where ANOVA was significant, letters following the traits indicate significant differences between treatments at ρ< 0.05. The same letters signify no significant differences between treatments. Where there were not significant differences in ANOVA, values were removed and the mean can be used for the trait.

CC, Canopy Cover; R, Trait from RGB sensor; L, Trait from LiDAR sensor; V, Trait from VNIR-hyperspectral sensor; RS, Row Segment; Y, Year.

† = ANOVA significance based on p-value: >0.05= NS,<0.05 = *,<0.01 = **,<0.001 = ***.

**Table 4:** Repeatability of remote sensing traits from RGB (red-green-blue), LiDAR (light detection and ranging), and VNIR (visible near infrared hyperspectral) sensors in maize experiments from 2020 and 2021.

	Vegetative (0 - 650 GDD)					Flowering (650 - 900 GDD)					Grain Filling (900+ GDD)				
	CC^R^	CC 10%^L^	Height 95%^L^	Plot Volume^L^	NDVI^V^	CC^R^	CC 10%^L^	Height 95%^L^	Plot Volume^L^	NDVI^V^	CC^R^	CC 10%^L^	Height 95%^L^	Plot Volume^L^	NDVI^V^
Mean	0.83	0.71	0.83	0.79	0.86	0.75	0.7	0.94	0.92	0.91	0.77	0.84	0.97	0.95	0.84
															
Maize 2020	0.85 a	0.81 a		0.82 a			0.86 a		0.89 b					0.94 b	0.77 b
Maize 2021	0.81 b	0.57 b		0.75 b			0.53 b		0.95 a					0.96 a	0.91 a
															
RS1234	0.87 a		0.86 a	0.82 a	0.89 a		0.8 a		0.94 a			0.9 a	0.98 ab	0.97 a	
RS23	0.86 ab		0.86 a	0.84 a	0.88 a		0.75 ab		0.94 a			0.9 a	0.98 a	0.97 a	
RS14	0.85 ab		0.83 a	0.78 a	0.89 a		0.75 ab		0.92 a			0.85 ab	0.97 bc	0.95 b	
RS1	0.78 c		0.83 a	0.79 a	0.9 a		0.66 bc		0.91 a			0.78 bc	0.94 d	0.94 c	
RS2	0.82 bc		0.86 a	0.84 a	0.86 a		0.63 bc		0.94 a			0.86 ab	0.98 ab	0.96 a	
RS3	0.82 abc		0.84 a	0.82 a	0.87 a		0.76 ab		0.93 a			0.88 a	0.98 a	0.96 a	
RS4	0.79 c		0.76 b	0.65 b	0.74 b		0.54 c		0.87 b			0.75 c	0.97 c	0.91 d	
															
Trim ANOVA	0.907 NS^†^	0.725 NS	0.812 NS	0.807 NS	0.879 NS	0.904 NS	0.74 NS	0.827 NS	0.923 NS	0.567 NS	0.951 NS	0.722 NS	0.792 NS	0.328 NS	0.937 NS
Year ANOVA	0.009 **	<0.001 ***	0.077 NS	0.029 *	0.058 NS	0.131 NS	<0.001 ***	0.072 NS	<0.001 ***	0.192 NS	0.081 NS	0.328 NS	0.938 NS	<0.001 ***	<0.001 ***
RS ANOVA	0.003 **	0.617 NS	0.035 *	0.003 **	<0.001 ***	0.134 NS	0.005 **	0.143 NS	0.023 *	0.569 NS	0.27 NS	0.005 **	<0.001 ***	<0.001 ***	0.851 NS
RS x Y ANOVA	0.342 NS	0.93 NS	0.59 NS	0.968 NS	<0.001 ***	0.262 NS	0.093 NS	0.967 NS	0.258 NS	0.585 NS	0.996 NS	0.756 NS	0.177 NS	0.202 NS	0.996 NS

Significant differences were declared using ANOVA between plot trimming (Trim) techniques, years (Y), row segments (RS), and interaction (RS x Y) effects. Where ANOVA was significant, letters following the traits indicate significant differences between treatments at ρ< 0.05. The same letters signify no significant differences between treatments. Where there were not significant differences in ANOVA, values were removed and the mean can be used for the trait.

CC, Canopy Cover; R, From RGB sensor; L, From LiDAR sensor; V, From VNIR-hyperspectral sensor; RS, Row Segment; Y, Year.

† = ANOVA significance based on p-value: >0.05= NS,<0.05 = *,<0.01 = **,<0.001 = ***.

The interaction effect between row segment and year was significant in sorghum for CC10 (*ρ*<0.05) at flowering stage and NDVI (*ρ*< 0.01) at flowering stage (**Supplemental Table 3**). The repeatability value of CC10 at flowering growth stage was best in RS1234 in 2018, RS1234 in 2019, and RS23 in 2020. The least repeatable value for CC10 at flowering was from RS4 in 2018, RS3 in 2019, and RS1 in 2020. While the interaction effect was significant for NDVI at flowering stage in sorghum, the most and least repeatable data came from RS1234 and RS4, respectively, in all years of the sorghum experiment. NDVI at the vegetative growth stage was the only trait with a significant (*ρ*< 0.001) row segment x year interaction in maize with RS4 in 2020 being worse than other row segment combinations in either year (**Supplemental Table 4**).

The row segments used to extract remote sensing data was a significant factor impacting repeatability for ten of the fifteen (five traits at three growth stages) remote sensing traits in sorghum (**Table 3**). Average repeatability across all remote sensing traits was 0.83 in RS1234, 0.82 in RS23, 0.79 in RS2, 0.79 in RS3, 0.77 in RS14, 0.72 in RS4, and 0.71 in RS1. The highest repeatability values were generally observed for RS1234, but repeatability for RS1234 was only significantly greater than RS23 for NDVI at flowering growth stage. Repeatability from RS14 was significantly lower than repeatability of RS1234 for nine of the fifteen remote sensing traits. Repeatability from a single outer row was significantly less repeatable than RS14 (outer rows) for eight of the fifteen traits for RS1 and six of the fifteen traits for RS4. Often, repeatability of traits measured in the inner row segments (RS2 and RS3) was significantly higher than repeatability of traits from the outer row segments (RS1 and RS4).

Row segment was a significant factor impacting repeatability for nine of the fifteen (five traits at three growth stages) remote sensing traits in maize (**Table 4**). Average repeatability across all remote sensing traits in all three growth stages was 0.90 in RS1234, 0.89 in RS23, 0.89 in RS3, 0.88 in RS14, 0.87 in RS2, 0.84 in RS1, and 0.83 in RS4. Repeatability of traits from RS1234 and RS23 were never significantly different, and repeatability of traits from RS14 was only significantly lower for PV at the grain filling stage. Repeatability of traits extracted from RS1 and RS4 (outside single rows) was significantly lower than repeatability of traits from RS1234 for seven and eight remote sensing traits, respectively, and significantly less repeatable than RS14 for four and eight remote sensing traits, respectively. Often, repeatability of traits from the inner row segments (RS2 and RS3) was significantly higher than traits measured in the outer row segments (RS1 and RS4).

### 3.2 Impact of row selection on predictive modeling

**Figure 3:**
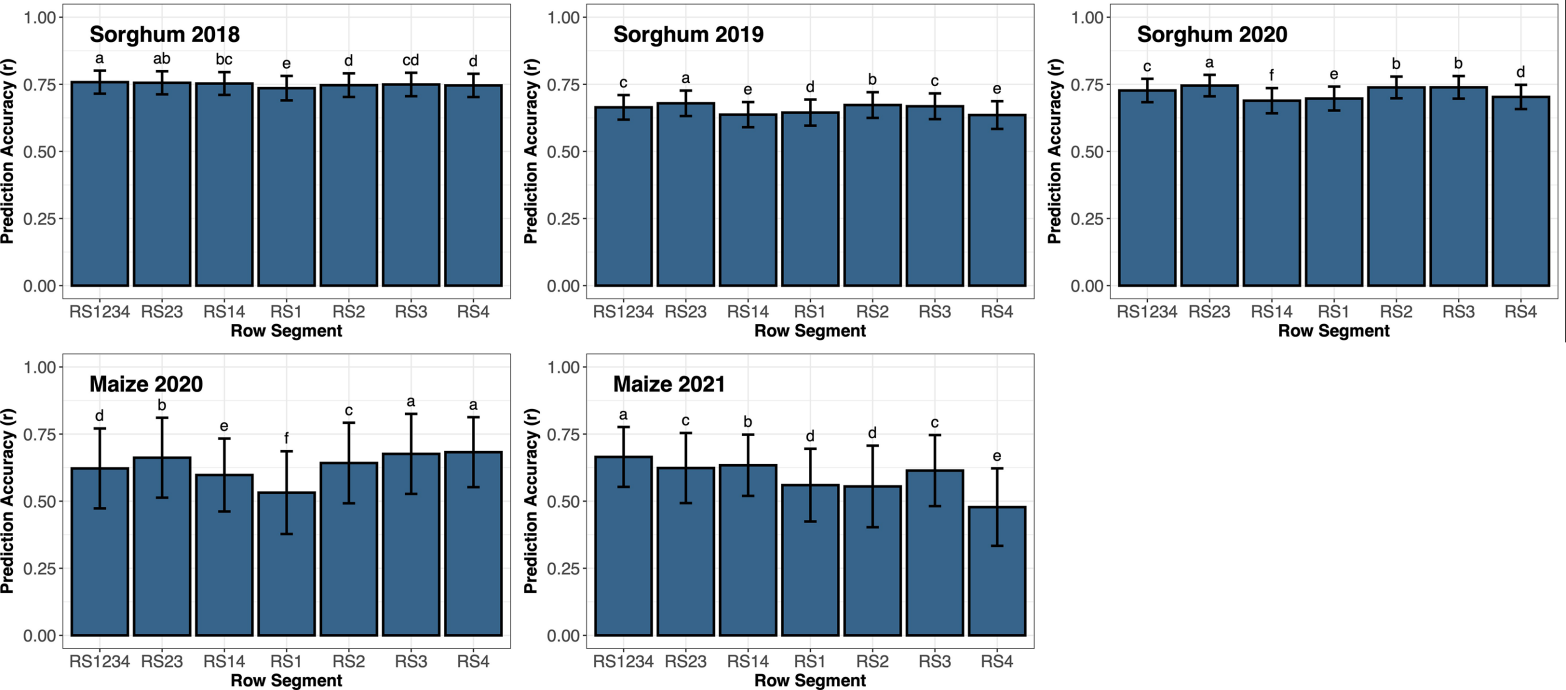
Prediction accuracy based on correlation (r) of support vector regression using all remote sensing traits from various row segments throughout the growing season to predict end-season biomass in sorghum or grain yield in maize. Error bars represent the standard deviation of the prediction accuracies. Letters above the bars represent significant differences between row segments using LSD (*ρ*< 0.05). Different letters represent significant differences between different row segments.

End-season sorghum biomass yield averaged 1,737 g m^-2^ (range: 745 to 3,989) in 2018; 1,720 g m^-2^ (range: 1,039 to 2,879) in 2019; and 1,746 g m^-2^ (range: 619 to 2,653) in 2020. The interaction effect between row segment and year was significant for both sorghum and maize. Thus, the effect of row segment was assessed for each year individually (**Figure 3**). Prediction accuracy of sorghum biomass ranged from 0.74 to 0.76 in 2018, 0.64 to 0.68 in 2019, and 0.69 to 0.75 in 2020. The highest prediction accuracy in 2018 used data from RS1234, though not significantly different from RS23. In 2019 and 2020, prediction accuracy using data from RS23 was significantly greater than all other row segment combinations. Remote sensing data from outer rows (RS14, RS1, or RS4) generally resulted in a significant decrease in prediction accuracy compared to remote sensing data including inner rows (RS23, RS1234, RS2, or RS3), except in 2018 where prediction accuracy from RS14 and RS23 and RS2, RS3, and RS4 were not significantly different.

Average maize grain yield was 10.9 Mg ha^-1^ (range: 3.2 to 14.8) in 2020 and 9.3 Mg ha^-1^ (range: 4.8 to 12.9) in 2021. Prediction accuracy of maize grain yield ranged from 0.53 to 0.68 in 2020 and 0.48 to 0.66 in 2021. Remote sensing data from RS3 and RS4 resulted in a prediction accuracy significantly higher than all other row segment combinations evaluated in 2020. Prediction accuracy using remote sensing data from RS23 was significantly greater than RS1234 which was significantly greater than RS14. The prediction accuracy of maize grain yield using data from RS1 was significantly lower than all other row segment combinations in 2020. However, prediction accuracy of maize grain yield in 2021 from RS1234 was significantly greater than all other RS combinations. Prediction accuracy from remote sensing data from RS14 was significantly greater than RS23. Prediction accuracy from RS3 was not significantly different from RS23, but it was significantly greater than RS1, RS2, and RS4. Remote sensing data from RS14 (outer rows) resulted in a significantly higher prediction accuracy when compared to RS1 and RS4 (single outer rows).

## 4 Discussion

### 4.1 Implications of row selection and plot trimming in remote sensing

Phenotyping capabilities have been the limiting factor to greater genetic gain in breeding programs based on the time consuming, laborious, costly, and often destructive nature of the endeavor (Cobb et al., 2013; Yang et al., 2017). Additionally, some traits, such as canopy cover, are difficult to quantitatively measure and are reduced to subjective estimates. High-throughput phenotyping through remote sensing can be used to produce large quantities of data on breeding plots that can be used *per se* or in end-season yield predictions to increase genetic gain in plant breeding. Key research questions related to processing of remote sensing data in breeding trials include the impact of row selection, plot trimming, and number of RS in a plot have not been quantitatively explored.

The border effect has been well documented in previous agronomic studies and has led to an emphasis on measurements of traits from the inner rows of multi-row plots (Bird, 1929; Genter, 1958; Gomez, 1972; Kramer et al., 1982; Bowman, 1989; Ceccarelli and Grando, 1996; Petersen, 1994; Reynolds – Braun, 2022) (**Supplemental Figure 1**). Nevertheless, border effect of remote sensing traits has been less documented. In this experiment, the border effect was evaluated based on repeatability and yield prediction accuracy using representative remote sensing traits related to geometric and chemistry-related responses (**Tables 3**, **4**; **Figure 3**). The impact of border rows can be observed by comparing all rows RS1234, inner rows (RS23), and outer rows (RS14) or comparing single inner rows (RS2 and RS3) with single outer rows (RS1 and RS4). Repeatability of remote sensing traits was often improved or not significantly different when considering whole plots rather than inner-plot rows. Prediction accuracy of end-season yield was often improved by considering just the inner-rows of a four-row plot. These results agree with Zhang et al. (2019) where soybean yield was best predicted when 20 to 80% of the plot area was used as it minimized border effect. Since yield was harvested from the inner rows in our study, it is possible that prediction accuracy from the inner-row segments was favorably biased and different results could have been obtained if yield had been determined using the harvest of all rows. Nevertheless, as yield is often harvested from the inner-plot rows in breeding experiments, this comparison has the most relevance. A follow-up study of interest could be to harvest each row of a plot individually to better associate yield and remote sensing data collection.

The significance of row selection indicated the presence of a border effect, which suggested that trimming the two ends of the plots could similarly impact results. Anderson et al. (2019) did not use any plot trimming technique. Malambo et al. (2017) trimmed 20 cm from the proximal and terminal ends of plots with a length 7.6 m in maize and 5.6 m in sorghum. Masjedi et al. (2018) trimmed 40 cm from the proximal and terminal ends of each plot of length 3 m in sorghum. Krause et al. (2019) trimmed 50 cm from the proximal and terminal ends of each plot in wheat experiments with plot lengths of 2.8 to 4 m. Tirado et al. (2020) created 20 bins within each row segment and evaluated the middle 12 bins for their analysis in maize with plot lengths of 3.65 m. While various methods have been used, none of these studies evaluated differences between plot trimming techniques. In our study, there was not a significant difference in repeatability values in all remote sensing data with and without 40 cm removed from the proximal and terminal ends of the plot boundary (**Tables 3**, **4**). The row selection grid in our experiment was created early in the growing season (V2-V4) when row segments were clearly visually separated. The plot segmentation process created segments that were defined by the end plants in each row. While plants continue to grow throughout the growing season, the grid remained unchanged. One hypothesis that explained the non-significant plot trimming effect was that plants grew outside of the bounding boxes developed early in the growing season, and this material was not included in the bounding box for trait measurements. Nevertheless, there is limited downside to plot trimming and it remains an effective measure to eliminate potential alley effect.

The number of row segments in a plot is an important consideration in experimental design constrained by the number of genotypes, replications, seed, and land area available. Generally, fewer row segments are used in the early stages of a breeding program to evaluate many genotypes and remove unfavorable genotypes based on highly heritable traits (i.e. disease resistance, plant height, etc.) (Acquaah, 2012). More row segments are used in advanced stages of a breeding program where yield performance is of primary concern. Nevertheless, remote sensing could enable researchers to evaluate plots with fewer row segments with greater accuracy and remove the necessity of plots with more row segments. In our study, RS1 and RS4 (single outer rows) and RS14 (outer rows) best represent one-row and two-row plots, respectively, as the border effect was present in these row segments. Remote sensing trait repeatability and prediction accuracy of either biomass in sorghum or grain yield in maize were generally improved as the number of RSs increased in both maize and sorghum. While this study only evaluated a maximum of four-row plots, it is likely that repeatability of remote sensing traits and prediction accuracy could be further increased using plots with more RS.

### 4.2 Implications and future work

The results of this study indicate that basic agronomic principles should be implemented to maximize the value of remote sensing data for plant breeding purposes. Plot trimming and excluding exterior rows should be used to limit the border effect from the alleys and neighboring plots. The number of RS in a plot is an important consideration when designing an experiment and should be increased when possible. Nevertheless, this experiment did not evaluate the tradeoff value of increasing the number of RS in a plot in comparison to increasing the number of replications in the experiment. Additionally, future studies should evaluate larger plot sizes extending beyond the four-row plot size used in this study. Finally, while plot trimming was not a significant factor in this study, it should be evaluated in experiments with different plot lengths or using other row segmentation techniques.

## 5 Conclusions

Remote sensing is a rapidly advancing area of phenomics enabling an increase in the amount of in-season data that can be evaluated. Within breeding programs, this data can be used *per se* or to predict end-season yield in the growing season. This study was performed to evaluate the importance of a border effect, plot trimming, and number of row segments used in remote sensing data in maize and sorghum. Generally, repeatability was improved when remote sensing data from more row segments was used in the analysis, and prediction accuracy was improved when excluding outer rows. While results in this study obtained using trimmed plots were not significantly different from when they were not trimmed, it should be considered in future studies to minimize any potential alley effect. Implementing these basic practices could help to maximize the value of remote sensing data and increase selection efficiency in a breeding program.

## Data availability statement

The original contributions presented in the study are included in the article/**Supplementary Material**, further inquiries can be directed to the corresponding author.

## Author contributions

MT conceived, initiated, and coordinated all of the experiments. MC, ED, and AH collected and processed the remote sensing data from the field trials. ST and NC analyzed the data and wrote the paper with contributions from the authors. All authors contributed to the article and approved the submitted version.
