# Supplementary material for: Row selection in remote sensing from four-row plots of maize and sorghum based on repeatability and predictive modeling
Source: Front Plant Sci. 2023 Jun 20;14:1202536. doi: 10.3389/fpls.2023.1202536 (PMC10318590; doi:10.3389/fpls.2023.1202536)
Supplement: Supplementary file 1 [file DataSheet_1.docx]

Supplementary Material

**Row Selection in Remote Sensing from Four-Row Plots of Maize and Sorghum Based on Repeatability and Predictive Modelling**

Seth A. Tolley, Neal Carpenter, Melba M. Crawford, Edward J. Delp, Ayman Habib, Mitchell R. Tuinstra^*^

*** Correspondence:** Mitchell R. Tuinstra, mtuinstr@purdue.edu

# Supplementary Figures and Tables

**Supplemental Figure 1.** Grain yield from each row segment of the maize experiment was obtained by harvesting rows individually in 2021. RS1234, RS23, and RS14 are the average of the rows collected. This supports previous work that repeatability is improved by using inner rows of larger plots with RS23 providing the most repeatable results.

**Supplemental Table 1.** Flight information over sorghum experiments in 2018, 2019, and 2020 highlighting when the data from each sensor was collected.

|  |  |  | **Sensor** | | |
| --- | --- | --- | --- | --- | --- |
| **Date** | **Crop** | **GDD** | **RGB** | **LiDAR** | **VNIR** |
| **20180604** | **Sorghum** | 308.8 | 1 | 1 |  |
| **20180611** | **Sorghum** | 383.2 | 1 |  |  |
| **20180620** | **Sorghum** | 508.2 | 1 | 1 |  |
| **20180625** | **Sorghum** | 566.3 |  |  | 1 |
| **20180627** | **Sorghum** | 591.5 | 1 |  |  |
| **20180702** | **Sorghum** | 666.2 |  | 1 |  |
| **20180710** | **Sorghum** | 771.5 | 1 | 1 |  |
| **20180711** | **Sorghum** | 784.3 |  |  | 1 |
| **20180718** | **Sorghum** | 874.6 | 1 | 1 |  |
| **20180725** | **Sorghum** | 958.4 |  |  | 1 |
| **20180801** | **Sorghum** | 1020.6 |  |  |  |
| **20180802** | **Sorghum** | 1032 |  |  | 1 |
| **20180806** | **Sorghum** | 1089.8 |  | 1 |  |
| **20190712** | **Sorghum** | 467 | 1 | 1 |  |
| **20190723** | **Sorghum** | 630 | 1 | 1 | 1 |
| **20190802** | **Sorghum** | 741.1 | 1 | 1 | 1 |
| **20190810** | **Sorghum** | 836.4 | 1 | 1 | 1 |
| **20190824** | **Sorghum** | 1011.1 | 1 | 1 | 1 |
| **20190905** | **Sorghum** | 1126.3 | 1 | 1 | 1 |
| **20200619** | **Sorghum** | 380.1 |  | 1 | 1 |
| **20200625** | **Sorghum** | 453.7 | 1 |  |  |
| **20200702** | **Sorghum** | 550.9 | 1 | 1 | 1 |
| **20200708** | **Sorghum** | 638.1 | 1 | 1 | 1 |
| **20200720** | **Sorghum** | 798.1 | 1 |  |  |
| **20200725** | **Sorghum** | 860.8 | 1 | 1 | 1 |
| **20200728** | **Sorghum** | 902.2 | 1 |  |  |
| **20200806** | **Sorghum** | 993.4 | 1 | 1 | 1 |
| **20200813** | **Sorghum** | 1072 | 1 | 1 | 1 |

**Supplemental Table 2.** Flight information over maize experiments in 2020 and 2021 highlighting when the data from each sensor was collected.

|  |  |  | **Sensor** | | |
| --- | --- | --- | --- | --- | --- |
| **Date** | **Crop** | **GDD** | **RGB** | **LiDAR** | **VNIR** |
| **20200617** | **Maize** | 355.4 | 1 | 1 | 1 |
| **20200625** | **Maize** | 453.7 | 1 |  |  |
| **20200702** | **Maize** | 550.9 | 1 | 1 | 1 |
| **20200708** | **Maize** | 638.1 | 1 | 1 | 1 |
| **20200717** | **Maize** | 752.5 | 1 | 1 | 1 |
| **20200720** | **Maize** | 798.1 | 1 |  |  |
| **20200725** | **Maize** | 860.8 | 1 | 1 | 1 |
| **20200728** | **Maize** | 902.2 | 1 |  |  |
| **20200806** | **Maize** | 993.4 | 1 | 1 | 1 |
| **20200813** | **Maize** | 1072 |  | 1 | 1 |
| **20200826** | **Maize** | 1224.4 |  | 1 | 1 |
| **20210610** | **Maize** | 208.2 | 1 |  |  |
| **20210616** | **Maize** | 285.2 |  | 1 | 1 |
| **20210617** | **Maize** | 293.5 | 1 |  |  |
| **20210622** | **Maize** | 363.5 | 1 |  | 1 |
| **20210702** | **Maize** | 487.5 |  | 1 | 1 |
| **20210703** | **Maize** | 497.2 | 1 |  |  |
| **20210719** | **Maize** | 703.1 | 1 |  |  |
| **20210723** | **Maize** | 751.4 |  | 1 |  |
| **20210727** | **Maize** | 810.8 | 1 | 1 | 1 |
| **20210802** | **Maize** | 880.5 | 1 |  |  |
| **20210808** | **Maize** | 950 | 1 | 1 |  |
| **20210816** | **Maize** | 1055.3 | 1 | 1 | 1 |
| **20210822** | **Maize** | 1137.5 | 1 |  |  |
| **20210828** | **Maize** | 1226.6 | 1 |  |  |
| **20210901** | **Maize** | 1282.7 |  | 1 | 1 |
| **20210906** | **Maize** | 1333.1 | 1 |  |  |
| **20210910** | **Maize** | 1371.1 |  | 1 | 1 |
| **20210917** | **Maize** | 1453.1 | 1 |  |  |
| **20210924** | **Maize** | 1520.3 |  | 1 |  |

Supplemental Figure 2. Illustration of 40 cm plot trimming on rows two and three in a four-row plot.

**Supplemental Table 3.** Repeatability of remote sensing traits with a significant row segment x year interaction in sorghum experiments from 2018, 2019, and 2020. Significant row segment x year interaction effects were declared using ANOVA. Where ANOVA was significant, letters following the traits indicate significant differences between treatments at $\rho$ < 0.05. The same letters signify no significant differences between treatments.

|  | **Flowering (650 - 900 GDD)** | |
| --- | --- | --- |
|  | **CC 10%^L^** | **NDVI^V^** |
| Sorghum 2018:RS1234 | 0.58 bc | 0.77 gh |
| Sorghum 2019:RS1234 | 0.59 abc | 0.85 c |
| Sorghum 2020:RS1234 | 0.68 ab | 0.9 a |
| Sorghum 2018:RS23 | 0.55 c | 0.75 i |
| Sorghum 2019:RS23 | 0.5 cde | 0.84 c |
| Sorghum 2020:RS23 | 0.7 a | 0.88 b |
| Sorghum 2018:RS14 | 0.45 def | 0.74 ij |
| Sorghum 2019:RS14 | 0.53 cd | 0.82 de |
| Sorghum 2020:RS14 | 0.52 cde | 0.87 b |
| Sorghum 2018:RS1 | 0.38 fg | 0.71 k |
| Sorghum 2019:RS1 | 0.38 fg | 0.78 gh |
| Sorghum 2020:RS1 | 0.41 efg | 0.82 ef |
| Sorghum 2018:RS2 | 0.54 c | 0.73 j |
| Sorghum 2019:RS2 | 0.43 efg | 0.81 f |
| Sorghum 2020:RS2 | 0.6 abc | 0.84 cd |
| Sorghum 2018:RS3 | 0.42 efg | 0.71 k |
| Sorghum 2019:RS3 | 0.36 g | 0.83 de |
| Sorghum 2020:RS3 | 0.68 ab | 0.85 c |
| Sorghum 2018:RS4 | 0.34 g | 0.64 l |
| Sorghum 2019:RS4 | 0.43 defg | 0.77 h |
| Sorghum 2020:RS4 | 0.45 defg | 0.79 g |
| RS x Y ANOVA | 0.014 * | 0.002 ** |

CC = Canopy Cover

L = From LiDAR sensor

V = From VNIR-hyperspectral sensor

RS = Row Segment

Y=Year

† = ANOVA significance based on p-value: >0.05= NS, <0.05 = *, <0.01 = **, <0.001 = ***

**Supplemental Table 4.** Repeatability of remote sensing traits with a significant row segment x year interaction in maize experiments from 2020 and 2021. Significant row segment x year interaction effects were declared using ANOVA. Where ANOVA was significant, letters following the traits indicate significant differences between treatments at $\rho$ < 0.05. The same letters signify no significant differences between treatments.

|  | **Vegetative (0 - 650 GDD)** |
| --- | --- |
|  | **NDVI^V^** |
| Maize 2020:RS1234 | 0.89 ab |
| Maize 2021:RS1234 | 0.89 ab |
| Maize 2020:RS23 | 0.88 ab |
| Maize 2021:RS23 | 0.88 ab |
| Maize 2020:RS14 | 0.89 ab |
| Maize 2021:RS14 | 0.89 ab |
| Maize 2020:RS1 | 0.93 a |
| Maize 2021:RS1 | 0.87 ab |
| Maize 2020:RS2 | 0.89 ab |
| Maize 2021:RS2 | 0.83 b |
| Maize 2020:RS3 | 0.85 b |
| Maize 2021:RS3 | 0.88 ab |
| Maize 2020:RS4 | 0.6 c |
| Maize 2021:RS4 | 0.88 ab |
| RS x Y ANOVA | <0.001 *** |

V = From VNIR-hyperspectral sensor

RS = Row Segment

Y=Year

† = ANOVA significance based on p-value: >0.05= NS, <0.05 = *, <0.01 = **, <0.001 = ***
